# Thermal and Calorimetric Evaluations of Polyacrylonitrile Containing Covalently-Bound Phosphonate Groups

**DOI:** 10.3390/polym10020131

**Published:** 2018-01-30

**Authors:** Svetlana Tretsiakova-McNally, Paul Joseph

**Affiliations:** 1 Belfast School of Architecture and the Built Environment, Ulster University, Newtownabbey BT37 0QB, Northern Ireland, UK; 2 Centre for Environmental Safety and Risk Engineering, Victoria University, P.O. Box 14428, Melbourne, VIC 8001, Australia; paul.joseph@vu.edu.au

**Keywords:** polyacrylonitrile, chemical modification, flame retardance, acrylic phosphonates, thermal degradation, combustion characteristics

## Abstract

One of the effective ways to enhance flame retardance of polyacrylonitrile (PAN) is through a reactive route, primarily developed in our laboratories, which involved chemical modification reactions utilising phosphorus-containing comonomers. In the present study, diethyl(acryloyloxymethyl)phosphonate (DEAMP) and diethyl(1-acryloyloxyethyl)phosphonate (DE1AEP) were synthesised and copolymerised with acrylonitrile (AN), under radical initiation in an inert atmosphere, in aqueous slurries. The thermal degradation and combustion characteristics as well as the extent of flame retardation were mainly assessed with the aid of various thermo-analytical and calorimetric techniques. It was found that the incorporation of phosphonate groups in polymeric chains of PAN resulted in improved flame-retardant characteristics. Furthermore, it was observed that the actual chemical environment of the phosphorus atom in the acrylic phosphonate modifying groups has little effect on the overall thermal degradation and combustion behaviours of the modified PAN systems. It was also observed that the predominant mode of flame retardance occurred in the condensed phase.

## 1. Introduction

Synthetic fibres encompass a wide variety of polymeric materials including polyolefins, acrylics, polyamides and polyesters. Polyacrylonitrile (PAN) is a well-established example of a fibre-forming polymer. Staple fibres based on PAN, being soft and resilient, are often used as substitutes or diluents for wool, and fabrics made from them often demonstrate good crease resistance and retention. Owing to their relatively high melting point (which often is accompanied by degradation) and significant carbon yields, PAN fibres are also considered to be the most suitable precursors of high-performance carbon fibres [1]. 

Similar to other organic polymeric materials, once heated the synthetic polymers will undergo various thermal and thermo-oxidative degradation processes that depend on the chemical nature of the fibre and on the intensity of the heat flux [2]. In most cases, a degrading synthetic polymeric substrate produces a variety of organic volatile compounds which, when mixed with the ambient air, form flammable mixtures. The size, shape and other morphological features of finished polymeric products have a strong influence on their overall flammability characteristics. For instance, owing to their large surface area to volume ratio, fibres are significantly more flammable than their moulded counterparts [3]. In addition, the burning behaviour of fabrics derived from fibrous materials is also influenced by a number of factors such as: the nature and intensity of the ignition source and time of its impingement; orientation of the fabric material and point of initiation of the ignition; ambient temperature and relative air humidity; velocity of air surrounding the material, etc.

When exposed to a heat source for a long period of time, PAN undergoes extensive degradation involving the production of combustible volatile compounds such as acrylonitrile, ammonia, other organic and inorganic nitriles as well as varying amounts of char; the latter strongly depending on the rate of heating [2]. Generally, acrylic fibres have limiting oxygen index (LOI) values of about 18, and burn quite readily accompanied by melting and sputtering. The mechanism of thermal degradation of PAN also strongly depends on the rate of heating. At low heating rates, intramolecular cyclization of side –C≡N groups is the main reaction pathway of PAN degradation, whilst at higher heating rates that are commensurate with those encountered in fires volatile-forming chain scission may prevail [1].

Successful strategies to reduce the flammability of synthetic polymers involve interrupting the complex combustion process, at one or more stages, to reduce the rate and/or to change the mechanism of combustion at that stage. From a practical point of view, this can be achieved by mechanical blending of a suitable flame-retardant compound with the polymeric substrate (i.e., by introducing an additive). Alternatively, this can be accomplished either by simple copolymerisation or by chemical modification of a pre-formed polymer (i.e., using a reactive component) [3]. Although the majority of the synthetic polymers are usually made more flame-retardant by incorporating additives, this strategy is often not very effective and could be problematic in the case of fibre-forming polymers, where deleterious effects on tensile properties, for example, may occur. In addition, a phase separation and/or a possibility of the additives leaching out are quite high during fibre production or equally plausible during the fibres’ life cycle, for example, within a knitted material. Recently, a layer-by-layer assembly technique was deployed to deposit fire retardant nanocoatings on to the surfaces of polymeric materials including acrylic fibres [4,5].

Acrylonitrile (AN) based polymers that are produced on an industrial scale are generally made flame-retardant through the use of additives (e.g., ammonium polyphosphate and decabromodiphenyloxide). The reactive route to flame retard PAN has also been accomplished by the incorporation of halogen-containing comonomers such as vinylidene chloride or bromide, vinyl chloride or bromide, and α-chloro- or α-bromo-acrylonitrile. The content of such comonomers in the modified systems can reach up to 15 mol % and they are often referred to as ‘modacrylics’. Although an adequate level of flame retardance can be achieved through this procedure, halogen-containing materials are perceived as threats to human and environmental health. From this point of view, phosphorus-containing fire retardants are often considered as relatively less toxic and more environmentally friendly alternatives [6,7]. For the past several years, we have been attracted to the alternative method of flame retarding synthetic fibres through chemical modification reactions, primarily, utilising a range of phosphorus- or phosphorus- and nitrogen-containing compounds [8,9]. There are other obvious advantages of this methodology such as: (a) only relatively low levels of modification are needed; (b) the fire-retardant groups are covalently attached to the main polymer chain and thus are more likely to be retained during the life cycle of the finished product; and (c) the resultant material is a copolymer, and hence the modification is more uniform along the polymer chains.

Currently, we are continuing our efforts to enhance flame retardance of PAN by deploying the reactive strategy. In this regard, we have previously reported on the improved flame retardance of AN-based polymers chemically modified with phosphorylamino and acrylic phosphate groups [8,10]. We have also employed pyrolysis combustion flow calorimetry (PCFC) as a useful screening tool that allowed the efficacies of the different modifying groups towards flame retarding some synthetic polymeric materials to be established [9]. The aim of the current work was to study the thermal/thermo-oxidative degradation behaviours and calorimetric characteristics of PAN chemically modified with phosphonate groups, where the penta-valent phosphorus present has slightly different chemical environments. Acrylic phosphonates can exert an influence on flame retardance “by virtue of either one or two effects: one arising from the backbone group and the other one from the pendant/side groups, and often the latter effect predominates” [7]. The corresponding reactive transformations were effected almost exclusively by copolymerisation reactions of AN with unsaturated acrylic phosphonates, carried out in aqueous slurry, at moderate temperatures using a redox initiation system. The recovered copolymers were purified, dried and subjected to a variety of spectroscopic, thermal and calorimetric techniques.

## 2. Materials and Methods

### 2.1. Materials

All chemicals, reagents and solvents were obtained from Sigma Aldrich (Gillingham, UK) with the exception of acryloyl chloride which was supplied by Alfa Aesar (Heysham, UK). AN was freed from small amounts of the inhibitor, 4-methoxyphenol, by passing it through a column packed with activated basic alumina. It was then stored in the dark, over molecular sieves (4 Å type), at 0–5 °C. The solvents and other reagents used in this study were purified, if necessary, according to the standard protocols published in the literature [11]. 

### 2.2. Preparation of Unsaturated Acrylic Phosphonates

Comonomers based on acrylic phosphonates, diethyl(acryloyloxymethyl)phosphonate (DEAMP) and diethyl(1-acryloyloxyethyl)phosphonate (DE1AEP), were synthesised by the condensation reactions of acryloyl chloride (AC) with diethyl(hydroxymethyl)phosphonate (DEHMP) and diethyl(1-hydroxyethyl)phosphonate (DEHEP), respectively, following the method of Liepins et al. [12]. The chemical structures and the degrees of purities of DEAMP and DE1AEP, as well as their precursors, were assessed with the aid of gas chromatography–mass spectrometry (GC–MS) analyses, and through ^1^H and ^31^P nuclear magnetic resonance (NMR) spectroscopy.

#### 2.2.1. Synthesis of Diethyl(acryloyloxymethyl)phosphonate (DEAMP)

**Scheme 1:**
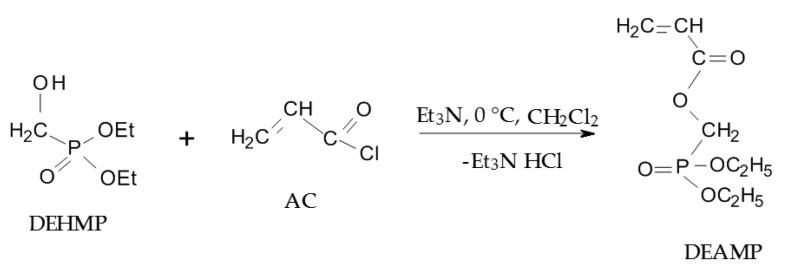
Synthetic route to diethyl(acryloyloxymethyl)phosphonate (DEAMP).

DEAMP was synthesised in a one-step procedure according to Scheme 1. Commercially supplied diethyl(hydroxymethyl)phosphonate (DEHMP: 117.69 g, 0.7 mol) and triethylamine (TEA: 98 cm^3^, 0.7 mol) were dissolved in anhydrous dichloromethane (DCM) (400 cm^3^), cooled in an ice bath and purged with argon for 30 min. To this solution acryloyl chloride (AC: 56.9 cm^3^, 0.7 mol) dissolved in anhydrous dichloromethane (DCM: 100 cm^3^) was added drop-wise, with stirring, for a period of 3 h. The reaction mixture was then warmed to room temperature, and subsequently stirred under argon atmosphere for further 65 h. The precipitated triethylamine hydrochloride was removed by filtration and the filtrate was washed with distilled water (5 × 50 cm^3^), and finally dried over magnesium sulphate for 24 h. The solvent was then removed by rotary evaporation to leave a pale yellow viscous liquid. Yield: 124 g, 80%. 

^1^H NMR (400 MHz, CDCl_3_, (δ ppm)): 6.38 [dd, 1H, H(**H**)C=CH(CO_2_–)]; 6.06 [dd, 1H, H_2_C=C**H**(CO_2_–)]; 5.76 [dd, 1H, H(**H**)C=CH(CO_2_–)]; 4.53 [d, 2H, –O–C**H**_2_P–]; 4.11 [m, 4H, –P–O–C**H**_2_–CH_3_]; 1.26 [t, 6H, –CH_2_–**CH**_3_]; ^31^P NMR (400 MHz, CDCl_3_, (δ ppm)): 18.81; GC/MS (CI): 13.81 min/[MH^+^]: *m*/*z* 223.

#### 2.2.2. Synthesis of Diethyl(1-acryloyloxyethyl)phosphonate (DE1AEP)

**Scheme 2:**
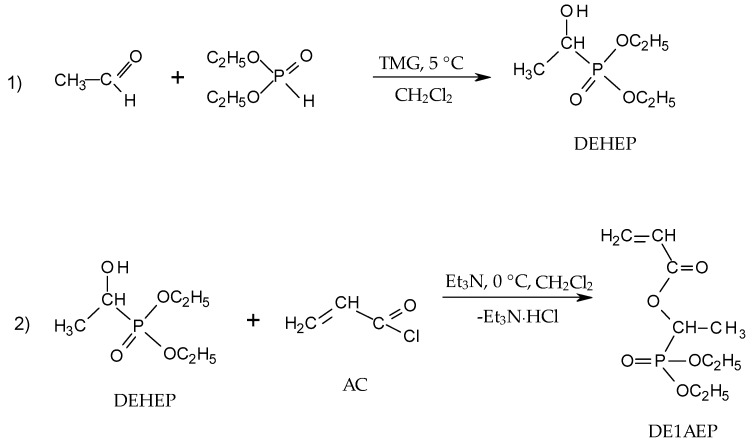
Synthetic route to diethyl(1-acryloyloxyethyl)phosphonate (DE1AEP).

DE1AEP was synthesised in two steps, as presented in Scheme 2.

● Step1: Synthesis of diethyl(1-hydroxyethyl)phosphonate (DEHEP)

47.1 g, (1.07 mol) of acetaldehyde, diethylphosphite (66.5 g, 0.48 mol) and DCM (200 cm^3^) were placed in a 500 cm^3^ round-bottomed flask connected to a water condenser, an argon inlet and a bubbler. This mixture was stirred, flushed with argon and cooled in an ice bath. About 1 cm^3^ of tetramethyl guanidine (TMG) was then added drop-wise to the reaction mixture. An exotherm was observed, and the temperature of the reaction mixture was kept below 5 °C with constant cooling. After the addition of TMG was complete, the reaction mixture was brought to room temperature and stirred for a further period of 2 h. The contents of the flask were washed with a single portion of saturated brine and the organic layer was separated and dried over sodium sulphate, filtered, and the volatiles were stripped by rotary evaporation to isolate DEHEP. Yield: 85 g (0.467 mol; near quantitative yield).

^1^H NMR (400 MHz, CDCl_3_, (δ ppm)): 5.33 [s, 1H, **H**O–CH(CH_3_)–]; 4.17 [m, 3H, **H_3_**C–CH(OH)–P–]; 4.14 [m, 1H, H_3_C–C**H**(OH)–P–]; 1.39 [m, 4H, –P–O–C**H**_2_–CH_3_]; 1.37 [t, 6H, –P–CH_2_–**CH**_3_]; ^31^P NMR (500 MHz, CDCl_3_, (δ ppm)): 27.30.

● Step 2: Esterification of DEHEP

DEHEP (18.2 g, 0.1 mol), TEA (13.1 g, 0.13 mol) and of dry DCM (200 cm^3^) were stirred in a 500 cm^3^ round-bottomed flask fitted with a double-walled water condenser and a bubbler, under argon, and the reaction mixture was cooled to 0 °C. To this mixture, AC (10.02 g, 0.11 mol) was added drop-wise via a pressure-equalizing dropping funnel under argon. After the addition was complete, the contents of the flask were warmed to room temperature and were allowed to react overnight under an argon blanket with stirring applied. The precipitated triethylamine hydrochloride was filtered and filtrate was washed with deionised water (4 × 50 cm^3^), dried over sodium sulphate, and finally the volatiles were stripped in a rotary evaporator to obtain DE1AEP. Yield: 19 g (0.08 mol); 80%.

^1^H NMR (400 MHz, CDCl_3_, (δ ppm)): 6.49 [dd, 1H, H(**H**)C=CH(CO_2_–)]; 6.20 [dd, 1H, H_2_C=C**H**(CO_2_–)]; 5.91 [dd, 1H, H(**H**)C=CH(CO_2_–)]; 5.39 [q, 1H, –O–C**H**–P–]; 4.22 [m, 4H, –P–O–C**H**_2_–CH_3_]; 1.72 [dd, 3H, d, –CH–C**H**_3_]; 1.35 [t, 6H, –CH_2_–C**H**_3_]; ^31^P NMR (500 MHz, CDCl_3_, (δ ppm)): δ 21.40; GC/MS (CI): 13.87 min/[MH^+^]: *m*/*z* 237.

### 2.3. Chemical Modification of Polyacrylonitrile (PAN)

**Figure 1:**
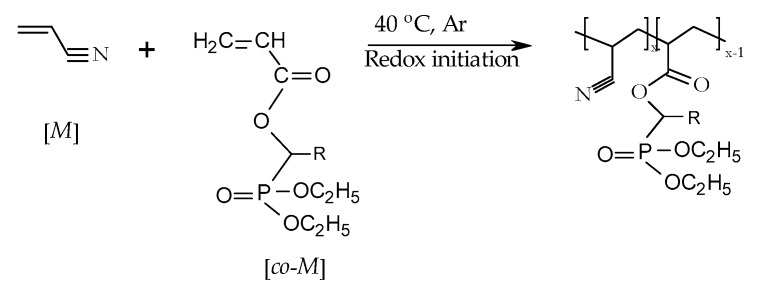
General scheme for copolymerisation of acrylonitrile (AN) with either DEAMP (when R = H) or with DE1AEP (when R = CH_3_).

Firstly, the homopolymer of AN, polyacrylonitrile (PAN), was synthesised in an aqueous slurry with the intention that this would be used as a control sample. In the current study, polymerisation of AN was initiated by the redox pair consisting of ammonium persulfate and sodium metabisulfite (Figure 1).

A typical synthetic procedure for the preparation of PAN or its copolymers by an aqueous slurry method is as follows: AN (13 cm^3^; 10.32 g), or a mixture of the monomer, AN, with the comonomers, DEAMP or DE1AEP, in a specified ratio was placed in a three-necked round-bottomed flask fitted with a magnetic stirrer, a water condenser and a bubbler containing deionised water (DIW) which had been previously flushed with argon and maintained at 40 °C. The mixture was then stirred for ca. 30 min with argon bubbling through it. Aqueous solutions of sodium metabisulfite and ammonium persulfate (each compound dissolved in 25 cm^3^ of DIW) were then added, one after the other, to the reaction mixture. The polymerisation reaction was then carried out for 16 h under argon. The resultant polymeric product was collected by filtration, and purified by repeated washings with DIW. The product was subsequently vacuum-dried at about 60 °C for several hours before further tests. Preparative data for the synthesis of PAN and corresponding copolymers are shown in Table 1.

### 2.4. Characterisation Techniques

GC–MS analyses were performed using an Agilent 6890N gas chromatograph coupled with Agilent 5973N mass spectrometer (Agilent Technologies, Manchester, UK). Agilent Technologies HP-Ultra 2 (25 m × 0.2 mm × 0.33 μm) column (Agilent Technologies, Manchester, UK) was used for the GC runs. Chemical ionization (CI) was employed using methane, and the spectra were scanned from 100–600 amu.

^1^H and ^31^P NMR spectra of P-containing reactants, monomers and polymers were recorded in solutions of deuterated chloroform (CDCl_3_) and deuterated dimethyl sulfoxide (*d*_6_-DMSO) on Bruker spectrometers (Bruker, Coventry, UK), operating at either 400 MHz for protons, or at 500 MHz instrument for phosphorus. The spectra were processed by employing a bespoke software after appropriate calibrations. 

Polymeric products were dissolved in *d*_6_-DMSO for the spectral analyses in solutions. The ^1^H NMR spectrum of each polymeric sample was examined closely to ensure the absence of the residual quantities of (co-)monomers in the final polymeric product. The recorded spectra were used to calculate the degree of phosphorus-containing groups that were incorporated into PAN chains. The general structure of the copolymers was considered as [*M*]*_x_*-[*co-M*]_(1−*x*)_, where *M* represents the monomeric units of AN with *x* as the mole fraction, and correspondingly *co*-*M* are the comonomeric units (either of DEAMP, or DE1AEP) with a mole fraction of (1 − *x*). Based on this, the mole fractions of the constituent monomeric units were calculated by taking the ratios of the integral areas of appropriately assigned signals in the ^1^H NMR spectra. Therefore, the content of *P* (wt %) in a copolymer was calculated from the following equation:

(1) $$\%P=\frac{(1-x)\cdot 31\cdot 100\%}{x\cdot M_{\left[M\right]}+(1-x)\cdot M_{\left[\mathrm{co-M}\right]}},$$

where M_[*M*]_ is a molecular mass of a monomeric unit *M*, i.e., AN; M_[*co-M*]_ is a molecular mass of a comonomeric unit *co-M*, as the case may be (i.e., DEAMP, or DE1AEP).

The thermo-gravimetric analyses (TGA) were carried out on ca. 10 mg samples with the aid of a Mettler Toledo TGA/SDTA851^e^ instrument (Mettler Toledo, Leicester, UK). The TGA runs were performed at a heating rate of 10 °C/min in the atmospheres of nitrogen, air and oxygen, at flow rates of 50 cm^3^/min, in the temperature interval between 30 °C and 700 °C. 

Pyrolysis combustion flow calorimetric (PCFC) measurements were performed on a Fire Testing Technology Ltd. micro-scale combustion calorimeter (Gosport, UK). The description of this method including the operating parameters is published in detail elsewhere [13]. The measured PCFC parameters including heat release capacity (HRC); peak heat release rate (PHRR); char residues; total heat release (THR); and temperature at PHRR (T_PHRR_), as well as the curves of heat release rate (HRR) vs. time are reported in the current study. For each run, an accurately weighed specimen (ca. 5 mg) of the sample ground to a fine powder was first heated to about 900 °C at a heating rate of 0.9 °C/s, in a stream of nitrogen (80 cm^3^/min), and the volatile species produced were further oxidised in air (20 cm^3^/min), in a chamber also maintained at 900 °C. The average values of three measurements for each sample are reported in this paper. Generally, the coefficients of variation for the values of PHRR, T_PHRR_, THR, and HRC did not exceed 5%, while those for char residues were not higher than 10%.

Bomb calorimetry measurements were performed on an IKA C200 instrument (IKA, Oxford, UK). Polymeric pelleted samples, weighing ca. 0.5 g, were placed inside a ‘bomb’ cell. The ‘bomb’ was filled with pure oxygen up to 30 bars of pressure, and subsequently ignited. The final calorific values were displayed by the instrument using built-in software. For each sample, triplicate runs were done to achieve better accuracy of the results.

## 3. Results and Discussion

### 3.1. Synthetic Approaches

In our study, the choice of DEAMP and DE1AEP as free-radically polymerisable comonomers was governed by the following factors: (a) synthetic procedures are fairly straightforward, thus providing relatively high yields of the final products: around 80 wt %; (b) ease of availability and modest costs of the reactants; (c) reasonably high hydrolytic stability of phosphonates; (d) relatively high reactivity in copolymerisation reactions. The chemical structures and the degrees of purities of DEHMP, DEAMP, DEHEP and DE1AEP, determined form GC–MS and NMR spectra, and the relevant data were in agreement with those previously published [14,15,16].

Similar to the data reported by us previously [8,9,10], the preparation of polymeric products of AN in aqueous slurries is a facile route, providing relatively high yields of copolymers, above 80 wt % (Table 1). Compositions of the copolymers (i.e., mole fractions of phosphorus-containing monomeric units within the polymeric chains) were effectively varied by changing the ratios of reacting comonomers in the feed, [*M*]/[*co*-*M*], and by keeping the reaction time constant (approximately 16 h). However different yields of resultant polymers were obtained (Table 1). The redox couple of sodium metabisulfite Na_2_S_2_O_5_ and ammonium persulfate (NH_4_)_2_S_2_O_8_ can provide at least four free radicals for the initiation of polymerisation [17]. The ratio of Na_2_S_2_O_5_/(NH_4_)_2_S_2_O_8_, which can affect both the molecular weight and the degree of oxidation of the products, was kept constant for all polymerisation reactions carried out in this study (Table 1). Generally, the final polymeric products appeared to be white, powdery solids with no obvious signs of discolouration, which indicated the absence of side reactions, such as intramolecular cyclization of pendant nitrile groups that might have led to extended conjugation and hence absorption of light.

### 3.2. Thermo-Gravimetric Analyses (TGA)

**Figure 2:**
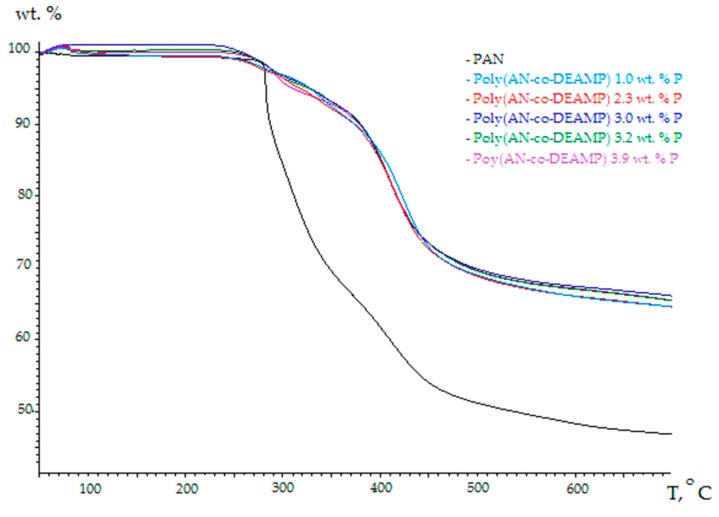
Thermo-gravimetric (TG) traces obtained under nitrogen for PAN and the copolymers poly(AN-*co*-DEAMP) containing different amounts of phosphorus: 1.0 (—); 2.3 (—); 3.0 (—); 3.2 (—); and 3.9 (—) wt % of P.

**Figure 3:**
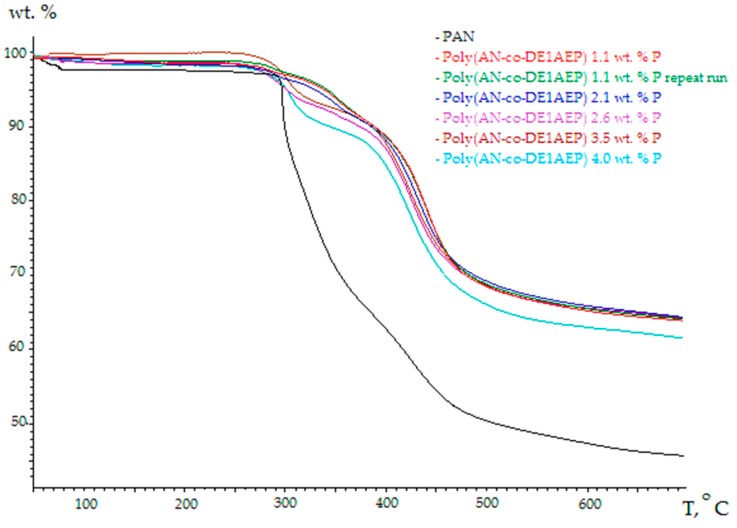
TG traces obtained under nitrogen for PAN and the copolymers poly(AN-*co*-DE1AEP) containing different amounts of phosphorus: 1.1 (—); 2.1 (—); 2.6 (—); 3.5 (—); and 4.0 (—) wt % of P.

The results of TGA showed that the incorporation of acrylic phosphonates into polymers of PAN significantly altered the TG profiles obtained in nitrogen, air and oxygen atmospheres. For example, thermograms of poly(AN-*co*-DEAMP) and poly(AN-*co*-DE1AEP), even with the minimal amounts of DEAMP, or DE1AEP, as the comonomeric units, recorded in nitrogen exhibited clear differences compared with those of homopolymer, PAN (Figure 2 and Figure 3). These results are similar to those reported for the PAN containing phosphorylamino and phosphate monomeric units [8,10]. In nitrogen, polymeric materials containing acrylic phosphonate moieties are characterised by a two-stage decomposition compared to a single-step process as observed for PAN (Figure 2 and Figure 3).

For a series of copolymers with various amounts of DEAMP, or DE1AEP, the temperatures corresponding to the onset of thermal degradation *T*_onset_, the slopes of the degradation steps and the amounts of char residue produced at 700 °C (in nitrogen, air and oxygen atmospheres) were significantly different to those recorded for PAN samples (Figure 2 and Figure 3; Table 2). As shown in Figure 2 and Figure 3, the copolymers containing phosphonate moieties start degrading at temperatures 10–50 °C lower compared to the homopolymer. The lower values of *T*_onset_ could be explained by an early thermal cracking of the modifying phosphonate groups [1,8,14]. The first weight losses observed for the copolymers are linked to the elimination of the ethylene molecules from the ethyl groups attached to the phosphorus atom (O=P–(O**C_2_H_5_**)_2_) and the subsequent formation of phosphoric acid species [1]. 

As follows from Table 2 and from the TG traces shown on Figure 2 and Figure 3, the copolymers of AN containing phosphonate groups generated significantly more char residue during the TGA runs as compared to PAN, in all three atmospheres. This trend was most pronounced in the case of the degradation of polymeric products under the oxygen atmosphere. Indeed, the incorporation of about 4.0 wt % of phosphorus into the polymeric chains leads to an almost 30-fold increase in the amount of char formed in oxygen at 700 °C. In the case of the copolymers, the amount of residue formed in nitrogen was on average 10–20% higher than in those formed in air. The percentages of the residues formed at 700 °C under nitrogen and air were found to increase as the content of phosphorus in the copolymers rose up to about 2.6–3.0 wt %, but with diminishing returns of the char yields thereafter (Table 2). 

TGA results, both in air and in oxygen, showed that, for all the copolymers, first mass losses occurred at temperatures lower than those observed in a nitrogen atmosphere. The corresponding TG traces obtained under air and oxygen can be found in Supplementary Materials. This can be attributed to the fact that the presence of oxygen accelerated thermal degradation processes. In addition, the TG residues obtained for the copolymers in an oxygen atmosphere appeared to be more resistant to further oxidation at 700 °C. This could be associated with better structural integrity and improved stability of the char residues, as well as with their more inert chemical nature that is less prone to further degradation. The presence of phosphoric acid species may also enhance the formation of a protective layer on the surface of char residues, thus preventing further oxidative reactions. In addition, the slightly different chemical environment of the P atom in DEAMP and DE1AEP groups seems have no significant effect on TGA results.

### 3.3. Pyrolysis Combustion Flow Calorimetric Evaluations

**Figure 4:**
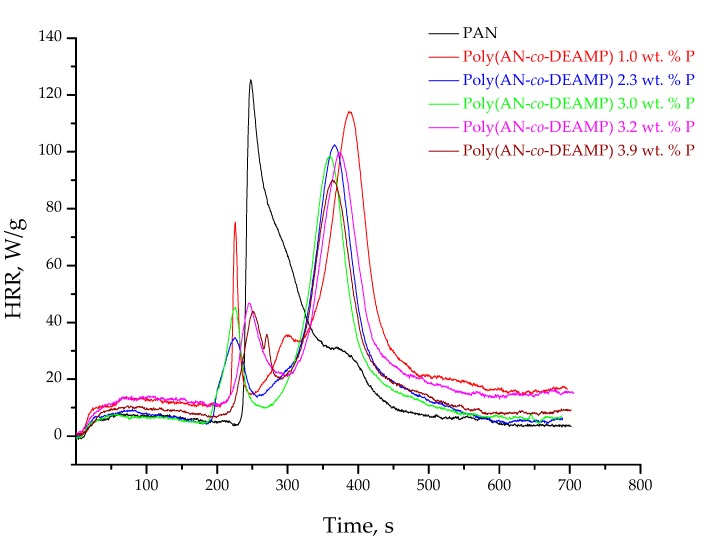

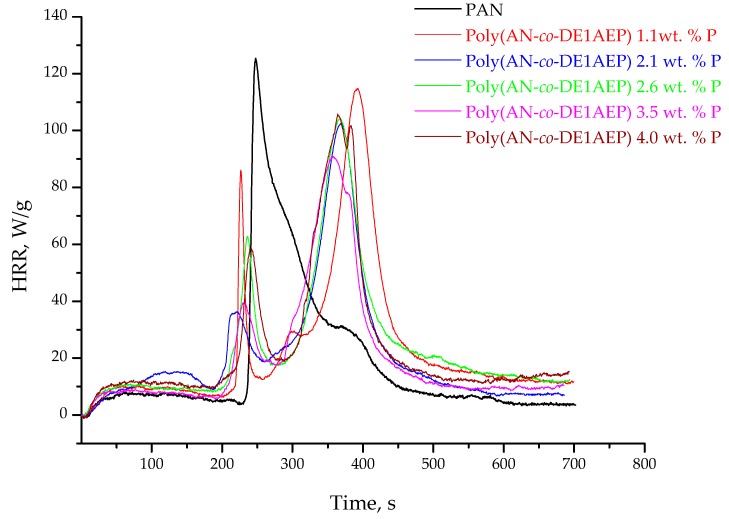
Heat release rate profiles for the samples of PAN and poly(AN-*co*-DEAMP) (**a**); PAN and poly(AN-*co*-DE1AEP) (**b**), containing various amounts of phosphorus.

We have already utilised the technique of pyrolysis combustion flow calorimetry (PFCF) to help establish the efficacies of the different P-containing groups towards flame retarding some chain-growth polymers [9]. In the current study, the PCFC data confirmed the flame-retardant effect of DEAMP and DE1AEP groups attached to the PAN chains. For example, the introduction of phosphonate units into the polymeric chains drastically changed the shape of HRR profiles (Figure 4, coloured curves). 

As follows from the plots shown in Figure 4, instead of one broad peak characteristic for PAN at around 290 °C, two peaks were observed in the case of the copolymers poly(AN-*co*-DEAMP) and poly(AN-*co*-DE1AEP). For a series of poly(AN-*co*-DEAMP) first peaks, either narrow or relatively small, were registered in the temperature range from 190–240 °C, whilst second peaks, higher and broader ones, were shifted to higher temperatures with the maxima registered at 406–429 °C. In the case of poly(AN-*co*-DE1AEP) first peaks were observed at 190–240 °C and second peaks—with maxima at 409–432 °C. In comparison to PAN, for the copolymers, the heights and the areas of the peaks were significantly reduced. Figure 4 also confirms that thermal degradation of copolymers started 10–50 °C prior to that of the unmodified polymer. A similar pattern was found for thermograms obtained in nitrogen, as discussed earlier.

The data summarised in Table 3 indicates that the reactive modification of PAN with acrylic phosphonate groups leads to the reduction in peak of the heat release rate. Indeed, for poly(AN-*co*-DEAMP) and poly(AN-*co*-DE1AEP) the PHRR values were reduced by a factor of 1.5–1.9. The lowest value of PHRR was recorded for the copolymer containing 0.089 mole fraction of DE1AEP groups (4.0 wt % P): 93.4 W/g. The HRCs, whose values are considered a reliable indicator of a material’s flammability, were also found to be significantly reduced for the modified systems [13]. For example, the incorporation of DEAMP (0.085 mole fraction) and DE1AEP (0.089 mole fraction) groups caused a decrease in the HRC values by almost 50%. It should be also noted that the modification of PAN, even with nominal amounts of DEAMP or DE1AEP, reduced the HRC by 61% and 66%, respectively. Therefore, it can be concluded that the incorporation of covalently-bound acrylic phosphonate groups into PAN chains changed its thermal degradation behaviour and resulted in an enhancement of flame retardance. It is interesting to note here that the THR values, which could indicate the total amount of fuel generated in PCFC runs, largely remained unchanged in the copolymers (Table 3). Once again, we have not observed a significant influence of phosphorus chemical environments on the PCFC data.

### 3.4. Mechanistic Aspects of Flame Retardance

The char yields recorded in PCFC tests, compared to the homopolymer, increased by 12–16 wt % for poly(AN-*co*-DEAMP) and by 8–15 wt % for poly(AN-*co*-DE1AEP). This trend correlates very well with the increase in the percentage of TG residues at 700 °C obtained in a nitrogen atmosphere (Table 2). This could be explained by the intramolecular cyclization reactions that are predominant at lower heating rates. For the modified polymers, the phosphoric acid species formed during the initial phase of degradation are able to function as nucleophilic centres, enhancing the intramolecular cyclization of the nitrile groups in the polymeric chains. A similar mechanistic pathway has been described for similarly modified systems in our previous works [7,14,18]. Thus, the condensed phase activity of the acrylic phosphonates in PAN chains, in enhancing the extent of intramolecular cyclization, leads to the increased levels char residues [1].

**Figure 5:**
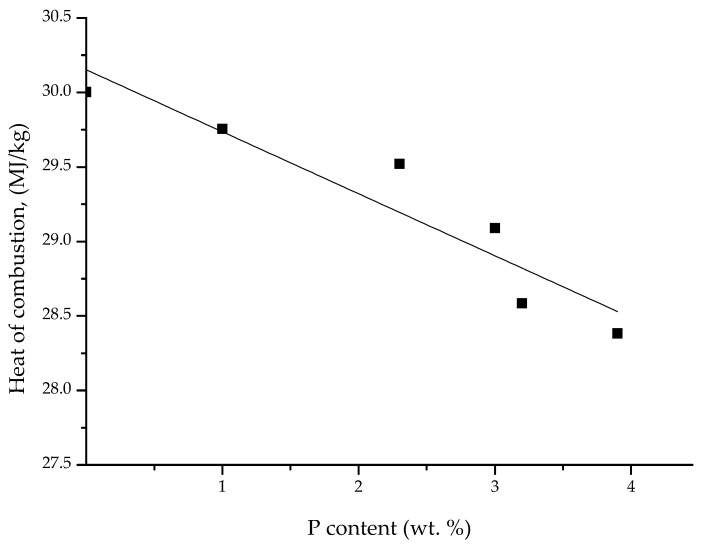
Heats of combustion versus content of phosphorus in poly(AN-*co*-DEAMP).

In addition to condensed phase activity of phosphonate moieties in PAN, there is a possibility of vapour phase activity emanating from volatile degradation products containing phosphorus [6]. This, in turn, could lead to the production of the higher amounts of carbon monoxide which is indicative of the lower combustion efficiencies. To verify this, we have measured calorific values, i.e., heats of combustion, for a series of copolymers of AN with DEAMP. The values of the heats of combustion were reduced for poly(AN-*co*-DEAMP) compared to PAN. Overall, the heats of combustion values were reduced almost linearly as the amount of covalently bound P in polymeric chains increased (Figure 5). This trend was observed by us previously in the systems of PAN modified with phosphorylamino groups [8]. Similar dependencies were observed in poly(AN-*co*-DE1AEP) systems but there was no noticeable difference compared to PAN containing DEAMP groups. 

## 4. Conclusions

The chemical modification of AN-based polymers by the incorporation of acrylic phosphonates, DEAMP or DE1AEP, resulted in significant improvements to their flame retardance, as gauged by increased TG and PCFC char yields and other measured combustion parameters, especially through the latter technique. It is assumed that the presence of P-containing groups will lead to an enhanced condensed-phase activity within overall flame-retardance activity. However, vapour-phase activities cannot be ruled out. In contrast to other modified systems studied by us in [8,10], there was no direct evidence that the chemical environment of the phosphorus atom in phosphonate moieties has a significant effect on thermal degradation and combustion behaviours. This reaffirms the previous observation that phosphonate groups are predominantly converted into acidic phosphoric species, thus triggering the carbonization process of the PAN chains and, therefore, the overall phosphorous loading is the governing factor and the slight differences in the chemical environment of the P-atom in the comonomers have very little influence on the prevailing flame-retardant mechanism(s) [1].

The current work opens up enormous opportunities to chemically modify AN-based polymer systems, made industrially through an aqueous slurry route, with a view to enhancing their flame retardance. Given that low levels of modification are only needed to bring about the desired level of flame retardance, it is expected that the thermo-mechanical properties and fibre-forming attributes of the modified polymers are not inferior in any way to the unmodified counterparts. The results obtained through the present work have also provided evidence of the mechanistic pathway to enhanced char formation in modified PAN systems; the initiating nucleophilic groups can be explored in more detail in the context of carbon-fibre production, using them as precursors.

## Figures and Tables

**Table 1:** Preparative data for syntheses of PAN, poly(AN-*co*-DEAMP) and poly(AN-*co*-DE1AEP).

Comonomer	[*M*] ^1^ (g)	[*co-M*] ^2^ (g)	Ratio [*M*]/[*co-M*] in the Feed (Mole Fraction)	Na_2_S_2_O_5_/(NH_4_)_2_S_2_O_8_	Water (cm^3^)	Yield (wt %)
-	10.32	-	1.00/0.00	2.86	290	88
DEAMP	10.24	0.90	0.98/0.02	2.86	290	86
DEAMP	10.00	1.78	0.96/0.04	2.86	290	86
DEAMP	9.84	2.68	0.94/0.06	2.86	290	89
DEAMP	9.68	3.58	0.92/0.08	2.86	290	80
DEAMP	9.37	4.48	0.90/0.10	2.86	290	85
DE1AEP	10.24	0.96	0.98/0.02	2.86	290	84
DE1AEP	10.00	1.88	0.96/0.04	2.86	290	85
DE1AEP	9.84	2.88	0.94/0.06	2.86	290	83
DE1AEP	9.68	3.78	0.92/0.08	2.86	290	81
DE1AEP	9.37	4.76	0.90/0.10	2.86	290	80

^1^ [*M*]—AN; ^2^ [*co-M*]—comonomer (DEAMP or DE1AEP).

**Table 2:** Characterisation data for PAN and the copolymers poly(AN-*co*-DEAMP) and poly(AN-*co*-DE1AEP).

Polymer System	[*co-M*], Mole Fraction	*P*, wt %	TG Residue at 700 °C in Nitrogen, wt %	TG Residue at 700 °C in Air, wt %	TG Residue at 700 °C in Oxygen, wt %
PAN	0	0	46.8	43.5	1.1
Poly(AN-*co*-DEAMP)	0.018	1.0	64.5	46.1	4.2
Poly(AN-*co*-DEAMP)	0.044	2.3	64.5	48.0	10.0
Poly(AN-*co*-DEAMP)	0.060	3.0	66.1	49.0	16.9
Poly(AN-*co*-DEAMP)	0.066	3.2	65.4	51.0	15.4
Poly(AN-*co*-DEAMP)	0.085	3.9	65.4	50.8	31.3
Poly(AN-*co*-DE1AEP)	0.020	1.1	65.3	50.4	2.6
Poly(AN-*co*-DE1AEP)	0.042	2.1	65.5	55.2	10.3
Poly(AN-*co*-DE1AEP)	0.052	2.6	65.5	49.7	15.1
Poly(AN-*co*-DE1AEP)	0.075	3.5	65.3	46.9	20.2
Poly(AN-*co*-DE1AEP)	0.089	4.0	62.6	45.5	31.0

**Table 3:** Pyrolysis combustion flow calorimetry (PCFC) parameters of PAN, poly(AN-*co*-DEAMP) and poly(AN-*co*-DE1AEP).

Polymer System	[*co-M*], Mole Fraction	*P*, wt %	*T*_PHRR_^1^, °C	PHRR, W/g	THR, kJ/g	HRC, kJ/g·K	PCFC Char Yield, wt %
PAN	0	0	289	182.7	14.9	200.7	45.6
Poly(AN-*co*-DEAMP)	0.018	1.0	429	112.2	17.5	121.9	58.4
Poly(AN-*co*-DEAMP)	0.044	2.3	417	102.3	13.6	110.9	57.1
Poly(AN-*co*-DEAMP)	0.060	3.0	412	99.1	14.8	107.2	58.7
Poly(AN-*co*-DEAMP)	0.066	3.2	409	99.0	16.1	107.5	57.1
Poly(AN-*co*-DEAMP)	0.085	3.9	406	93.5	14.4	101.5	61.2
Poly(AN-*co*-DE1AEP)	0.020	1.1	432	121.8	16.8	131.7	55.8
Poly(AN-*co*-DE1AEP)	0.042	2.1	418	103.3	15.0	112.0	53.8
Poly(AN-*co*-DE1AEP)	0.052	2.6	412	96.7	15.9	105.5	60.1
Poly(AN-*co*-DE1AEP)	0.075	3.5	409	96.9	14.7	105.3	56.2
Poly(AN-*co*-DE1AEP)	0.089	4.0	411	93.4	15.5	102.1	56.8

^1^ T_PHRR_ related to the first peaks on heat release rate (HRR) vs. time curves are not reported here.

## Supplementary Materials

The following are available online at http://www.mdpi.com/2073-4360/10/2/131/s1, Figure S1: TG traces obtained under air for PAN and the copolymers poly(AN-*co*-DEAMP) containing different amounts of phosphorus, Figure S2: TG traces obtained under oxygen for PAN and the copolymers poly(AN-*co*-DEAMP) containing different amounts of phosphorus, Figure S3: TG traces obtained under air for PAN and the copolymers poly(AN-*co*-DE1AEP) containing different amounts of phosphorus, Figure S4: TG traces obtained under oxygen for PAN and the copolymers poly(AN-*co*-DE1AEP) containing different amounts of phosphorus.
