# Supplementary material for: Thermal and Calorimetric Evaluations of Polyacrylonitrile Containing Covalently-Bound Phosphonate Groups
Source: Polymers (Basel). 2018 Jan 30;10(2):131. doi: 10.3390/polym10020131 (PMC6414975; doi:10.3390/polym10020131)
Supplement: Supplementary file 1 [file polymers-10-00131-s001.pdf]

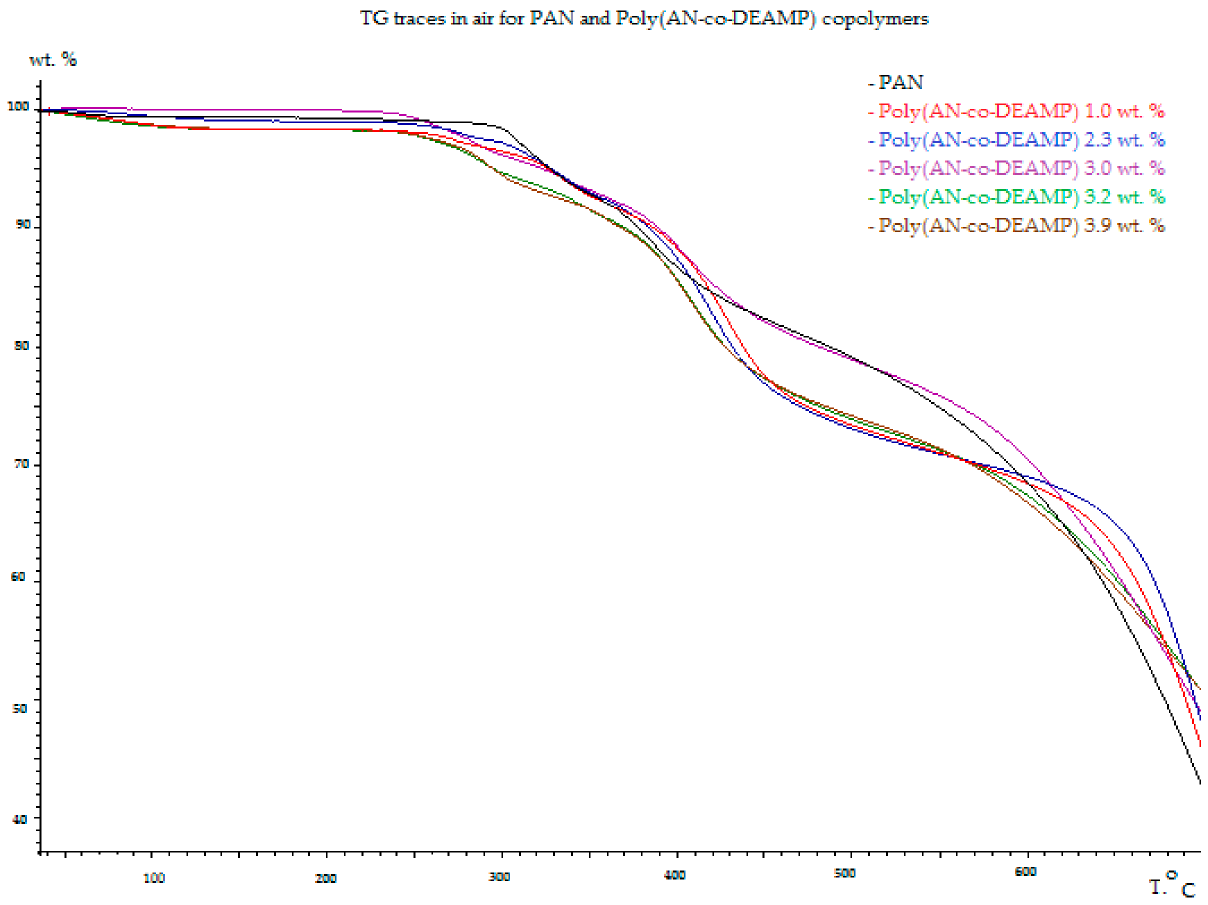

Figure S1. TG traces obtained under air for PAN and the copolymers poly(AN-*co*-DEAMP) containing different amounts of phosphorus.

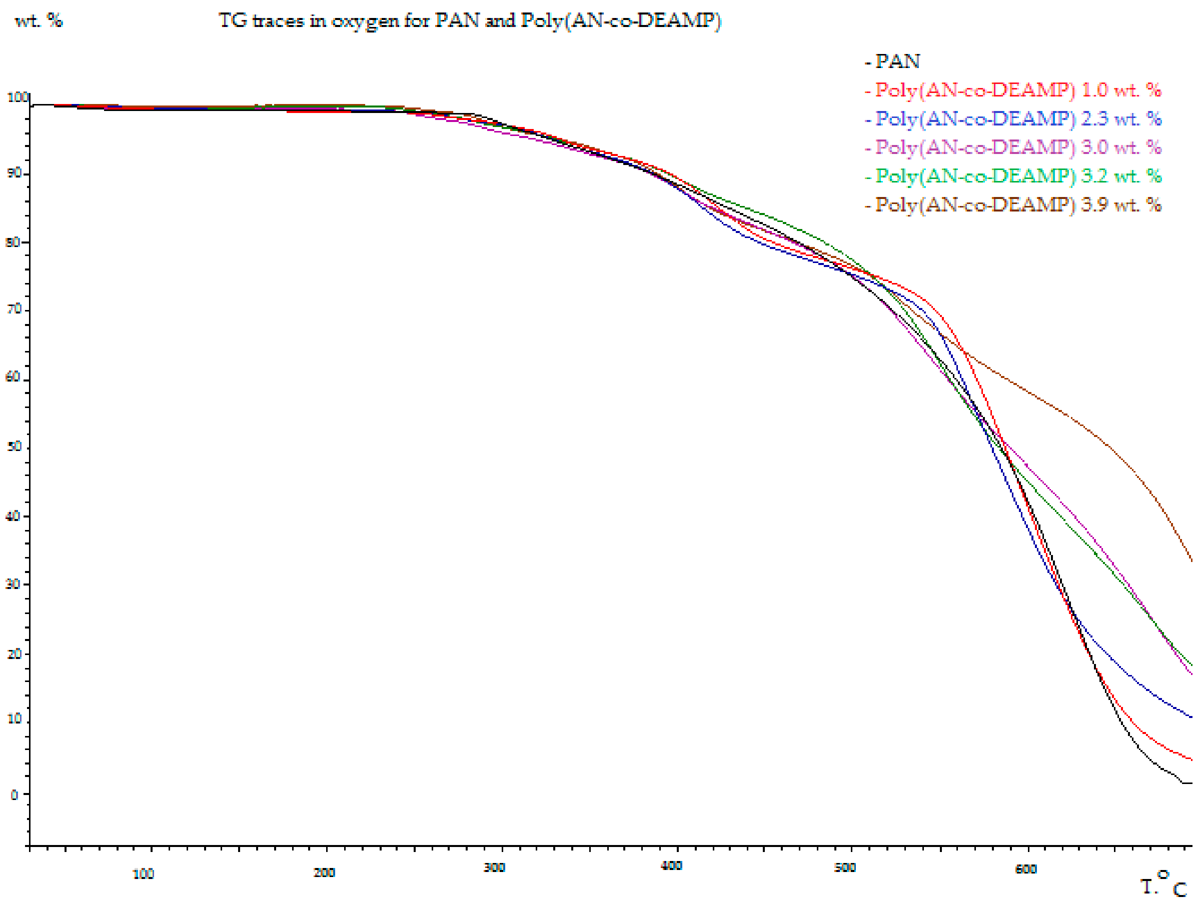

Figure S2. TG traces obtained under oxygen for PAN and the copolymers poly(AN-*co*-DEAMP) containing different amounts of phosphorus.

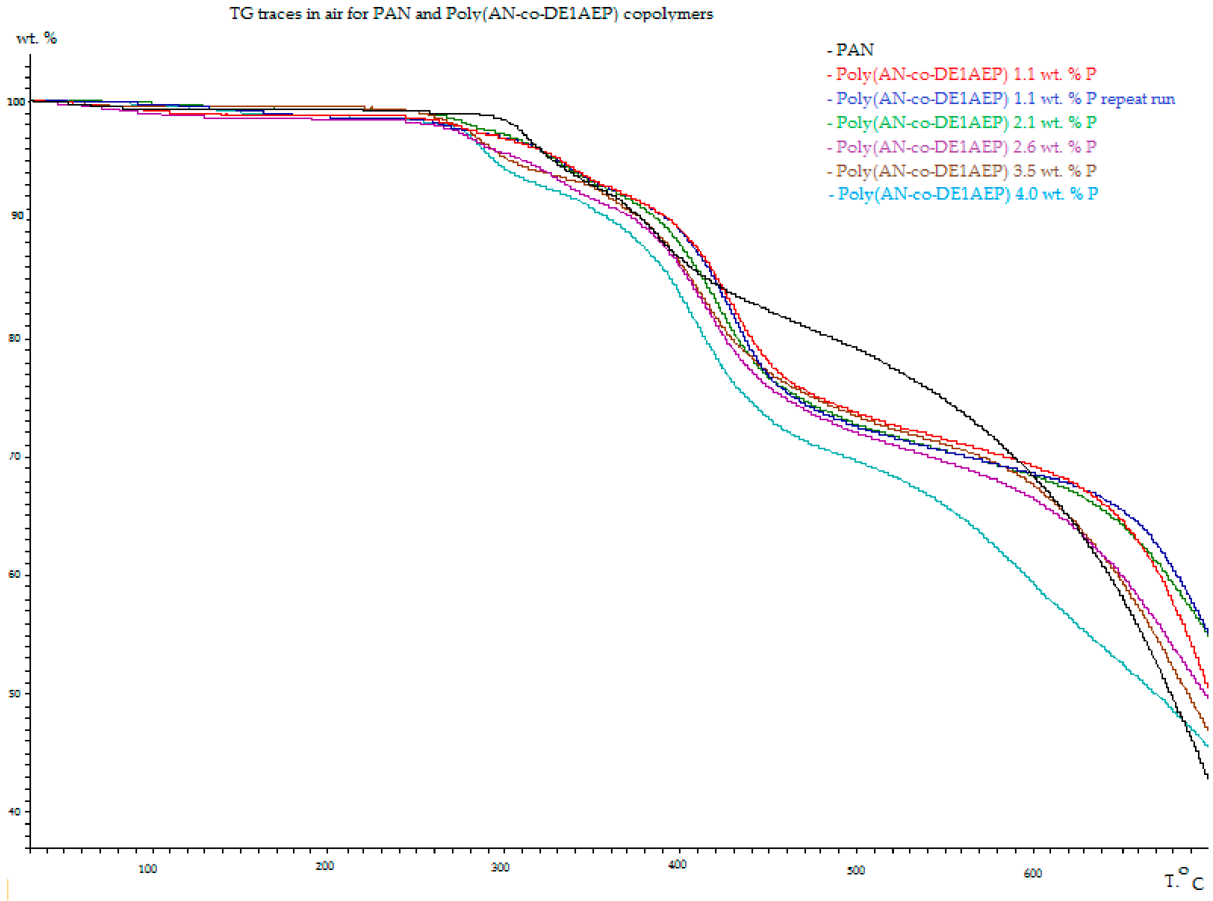

Figure S3. TG traces obtained under air for PAN and the copolymers poly(AN-*co*-DE1AEP) containing different amounts of phosphorus.

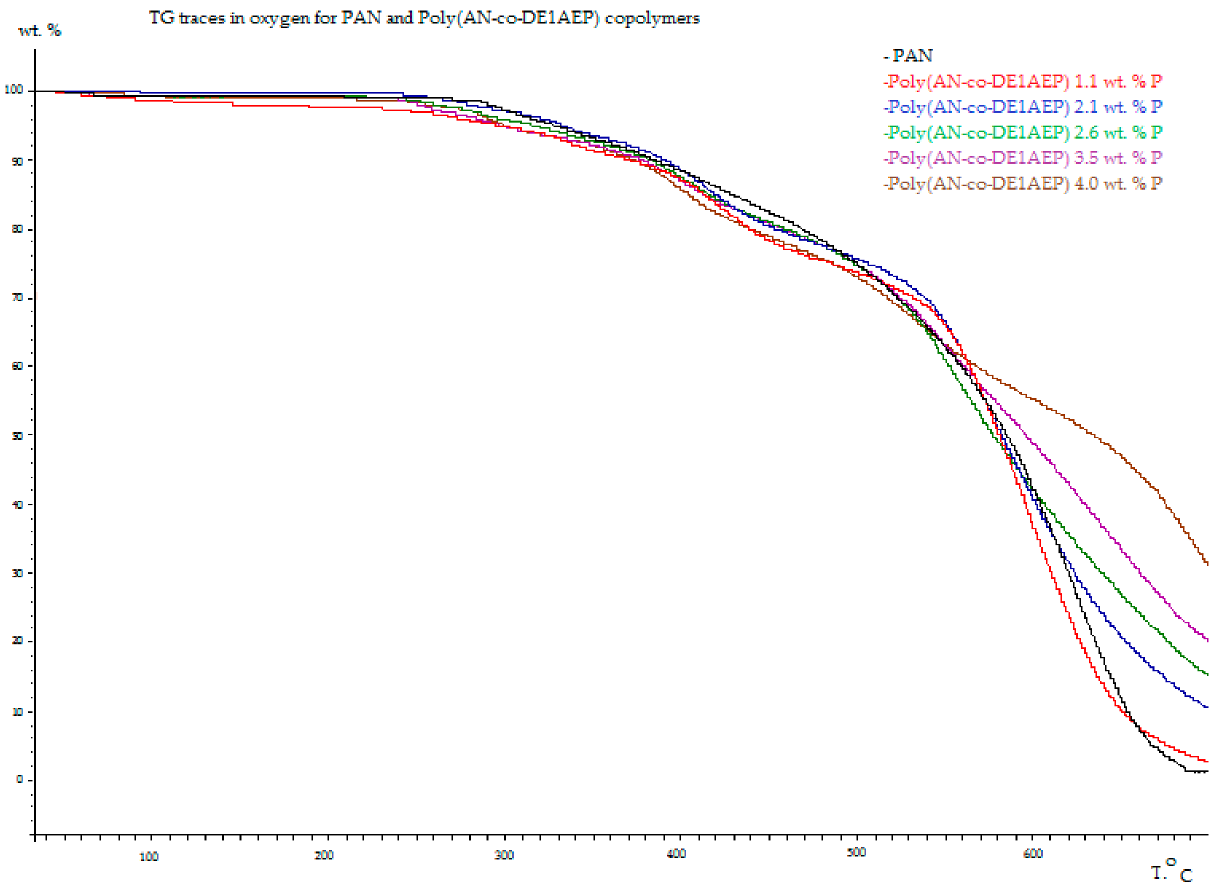

Figure S4. TG traces obtained under oxygen for PAN and the copolymers poly(AN-*co*-DE1AEP) containing different amounts of phosphorus.
